# Gases in Modern Warfare

**Published:** 1918-08

**Authors:** 


### MEDICINE

**Gases in Modern Warfare.** Editorial *Journal of the American Medical Association,* April 20, 1918.

Despite the fact that the daily newspapers frequently report the use of poisonous gases in the conduct of the war on the European continent, few physicians understand much more about the methods employed, the substances used, the protections devised and the untoward effects encountered than does the layman who carefully follows the reports from the centers of conflict. After all, this is not strange. Gas warfare is an innovation in the struggle between armies. It seemed so unlikely in 1914 that any enemy would introduce this atrocious form of attack that when the first reports of the preparation to employ it reached the allied forces they were scarcely credited, and no serious notice was taken of the information. With the initiation of the first gas attacks by the Germans in 1915, modern modes of conducting war were radically modified.

The story of the varied types of this hellish performance, of how the unexpected was met and mastered, of the frequently altered forms and phases of gas warfare conducted with the aid of skilled engineers, technologists, and chemists, has scarcely traveled beyond the trenches. It has been full of surprises, replete with difficulties such as confront the student of new diseases, and, if we may forget the awful horror of the consequences, the use of gases has formulated questions of broad scientific import. However terrifying the prospects may be, we must realize that a new and deadly weapon has been used, and its entrance into the arena must be reckoned with by the application of skill and knowledge.

What is the deadly gas, and how is it used? To this the answer may be made that there already are literally a score of harmful gases used in more than one way that have attained some notoriety[1]. First used and best recognized in medical circles was chlorin gas. This is sent out as a cloud from cylinders concealed beneath the parapets. The wind must be of suitable velocity and direction to make the attack effective. Protection is afforded by a respirator

1. The basis for this statement and others pertaining to the subject is found in the report of an interesting lecture by Major S. J. M. Auld of the British Military Mission, as published in the *Journal o the Washington Academy of Science*, 1918, 8, No. 3.

which causes the gas-laden air breathed to pass through an absorbent soaked in solutions of alkali carbonate or thiosulphate. The next surprise came in the form of carbonyl chlorid, or phosgen ($COCl_2$), an insidious gas difficult to protect against. Fortunately, a partial protection was soon found in helmets saturated with sodium phenate. It became apparent, to quote a military expert, that three things really matter in gas warfare, and these were all emphasized at this early period. They are (1) increased concentration of the gases used, so that the protective devices no longer suffice; (2) surprise attacks, whereby the troops are gassed before adequate provision for protection can be made; and (3) the use of unexpected new materials.

Accordingly, when carbonyl chlorid began to be used by the Germans in increased concentration, further provision to meet this became necessary. Here chemical ingenuity suggested the use of hexamethylenamin in conjunction with sodium phenate. On the horror of gas clouds the frightfulness of gas shells presently was superimposed. They are asserted to be the most important of all methods of using gas, and are still in the course of development. The widely proclaimed " tear " gases were delivered in this fashion. Originally xylyl bromid or benzyl bromid, obtained by bromination of the higher fractions of coal-tar distillates, formed the contents of the " tear " shells. A concentration of some of these lacrimators in the proportion of one part in a million makes the eyes water severely. The enormous extent to which such chemical products have been employed is indicated by the fact that the Germans have put down heavy barrages of " tear " gas shells. In 1916, such highly poisonous substances as trichloromethyl-chloroformate were included, the attempt often being made to increase the concentration by the mode of shelling so that some of the gas would pass through the protective helmets, as it actually did at times. Hence we can understand Auld's contention regarding the significance of suitable protective devices. " Respirators, " he says, " have to fulfil two requirements which are quite opposed to one another. In the first place they should be sufficiently large and elaborate to give full protection against any concentration of any gas, whereas military exigency requires that they be light and comfortable. It is necessary to strike a balance between these two. Upon a proper balance depends the usefulness of the respirator. Oxygen apparatus will not do on account of its weight and its limited life. Two hours' life is excessive for that type. The side that can first force the other to use oxygen respirators for protection has probably won the war. "

Last summer witnessed the beginning of the use of a still more harassing gas. An added lacrimator in the form of phenyl carbylamin chlorid has been tried; then came the celebrated "sneezing" gas, diphenyl-chloroarsin, intended to make a soldier sneeze so badly that he will be unable to keep his mask on. But the surprise that scored heavily in 1917 was the "mustard gas," dichlorodiethylsulphid. As many as 50,000 shells containing this "superlacrimator" have been reported as fired in a single night's bombardment. The effect is most insidious. The gas has a distinctive smell, rather like garlic than mustard. It has no immediate effect on the eyes, beyond a slight irritation. After several hours the eyes begin to swell and inflame and practically blister, causing intense pain, the nose discharges freely, and severe coughing and even vomiting ensue. Direct contact with the spray causes severe blistering of the skin, and the concentrated vapor penetrates the clothing. The respirators, of course, do not protect against this blistering.

In passing, we may recall the German hand grenades filled with bromin, chloracetone, chlorsulphonic acid, sulphur trioxid or dimethyl sulphate — surely a ghastly array! If the "colorless, odorless, invisible and highly poisonous" gas had not yet come to the front, its near relatives have nevertheless been in evidence. MEANWHILE, THE CHEMICAL LABORATORIES OF THE ALLIES HAVE NOT BEEN IDLE.

